# Cysteine-rich 61 (Cyr61): a biomarker reflecting disease activity in rheumatoid arthritis

**DOI:** 10.1186/s13075-019-1906-y

**Published:** 2019-05-21

**Authors:** Yong Fan, Xinlei Yang, Juan Zhao, Xiaoying Sun, Wenhui Xie, Yanrong Huang, Guangtao Li, Yanjie Hao, Zhuoli Zhang

**Affiliations:** Department of Rheumatology and Clinical Immunology, Peking University First Hospital, No.8, Xishiku Street, West District, Beijing, 100034 China

**Keywords:** Rheumatoid arthritis, Cysteine-rich protein 61, Biomarker, Disease activity, Treatment response

## Abstract

**Background:**

Numerous preclinical studies have revealed a critical role of cysteine-rich 61 (Cyr61) in the pathogenesis of rheumatoid arthritis (RA). But there is little literature discussing the clinical value of circulation Cyr61 in RA patients. The aim of our study is to investigate the serum Cyr61 level and its association with disease activity in RA patients.

**Methods:**

A training cohort was derived from consecutive RA patients who visited our clinic from Jun 2014 to Nov 2018. Serum samples were obtained at the enrollment time. To further confirm discovery, an independent validation cohort was set up based on a registered clinical trial. Paired serum samples of active RA patients were respectively collected at baseline and 12 weeks after uniformed treatment. Serum Cyr61 concentration was detected by enzyme-linked immunosorbent assay. The comparison of Cyr61 between RA patients and controls, the correlation between Cyr61 levels with disease activity, and the change of Cyr61 after treatment were analyzed by appropriate statistical analyses.

**Results:**

A total of 177 definite RA patients and 50 age- and gender-matched healthy controls were enrolled in the training cohort. Significantly elevated serum Cyr61 concentration was found in RA patients, demonstrating excellent diagnostic ability to discriminate RA from healthy controls (area under the curve (AUC) = 0.98, *P* <  0.001). In addition, the Cyr61 level in active RA patients was significantly lower than that in patients in remission/low disease activity, and it was inversely correlated with composite disease activity scores and almost all of the components in statistic. Further study in the validation cohort (*n* = 77) showed a significant increase of the Cyr61 level at 12 weeks in ACR responders (ACR20/50/70), while no significant change of the Cyr61 level from baseline was observed in non-responders.

**Conclusions:**

Serum Cyr61 levels were remarkably increased in RA patients compared with those in healthy controls. The Cyr61 concentration was inversely correlated with RA disease activity and upregulated in those therapeutic responders.

**Trial registration:**

Combination Therapy Prevents the Relapse of RA, NCT02320630. Registered 19 December 2014,

**Electronic supplementary material:**

The online version of this article (10.1186/s13075-019-1906-y) contains supplementary material, which is available to authorized users.

## Introduction

Rheumatoid arthritis (RA) is a chronic inflammatory autoimmune disease that causes progressive articular damage, functional loss, and comorbidity [1]. Although the development of effective biologics and small-molecule kinase inhibitors in the past two decades has substantially improved clinical outcomes of RA, a considerable number of patients respond insufficiently to the therapy [2]. Until now, the exact pathological processes involved in RA remain incompletely understood. Thus, we still need to investigate the pathological mechanisms and look for the appropriate markers to reflect disease activity as well as predict treatment response.

Cysteine-rich 61 (Cyr61, also called CCN1), a novel secreted matricellular protein, is encoded by a growth factor-inducible immediate-early gene. Cyr61 expression is at low levels in most adult tissues under homeostatic conditions, and remarkably elevated with the induction of cytokines and growth factors [3]. Numerous studies have revealed that Cyr61 played critical roles in cardiovascular development, inflammation, injury repair, and cancer. In RA, it was first reported by Haas et al. that Cyr61 mRNA was strongly increased in lymphoblastoid B cell lines derived from RA discordant monozygotic twins, being one of the three most overexpressed genes [4]. An increasing number of studies found elevated expression of Cyr61 protein in fibroblast-like synoviocytes (FLS), synovial fluid, and peripheral blood mononuclear cells from RA patients [5, 6]. Preclinical studies discovered that Cyr61 played a pivotal role in IL-17-dependent proliferation of FLS in RA and blocking Cyr61 with the specific neutralizing antibody ameliorated the inflammation of the collagen-induced arthritis mice in vivo [7, 8]. In addition, it was found that Cyr61 could not only stimulate IL-6 production by FLS via the Cyr61/αvβ5/Akt/NF-κB signaling pathway [8], but also promote neutrophil infiltration via upregulation of IL-8 production in RA-FLS [9]. A recent study demonstrated Cyr61 promoted vascular endothelial growth factor expression in osteoblasts through negative regulation of miR-126 via the PKC-α signaling pathway and increased endothelial progenitor cell angiogenesis in RA [10]. But the role of Cyr61 is paradoxical. In inflammatory liver diseases, Cyr61 contributed to myeloid-derived suppressor cell activation and expansion which served as negative regulators of excessive T cell responses [11]. Moreover, Cyr61 could strongly inhibit immune cell migration [12] and process anti-osteoclastogenic [13] and anti-inflammatory properties [14]. It is noteworthy that emerging preclinical studies have discussed the important roles of Cyr61 in RA pathogenesis, but there is little literature discussing the clinical value of circulation Cyr61 in RA patients. Therefore, in this study, we aim to look at the serum Cyr61 levels in RA patients in comparison with healthy controls and to investigate the correlation between serum Cyr61 level with clinical disease activity in our training cohort and validation cohort.

## Methods

### Study population

In this study, a training cohort was used for discovery and to identify some relationships, while an independent validation cohort was set up to further confirm. The training cohort was derived from consecutive RA patients who visited the Rheumatology clinic, Peking University First Hospital, China, from Jun 2014 to Nov 2018. All patients were > 18 years of age and satisfied the 2010 American College of Rheumatology (ACR) classification criteria for RA [15]. Serum samples were collected at the time of enrollment.

To further confirm discovery, a validation cohort consisted of RA patients from the screening phase of our prospective randomized and controlled clinical trial (ClinicalTrials.gov identifier: NCT02320630). Briefly, the aim of this trial was to compare the efficacy and pharmacoecomonic between combined conventional synthetic DMARDs and tumor necrosis factor (TNF) inhibitor in preventing relapse of RA. Active RA patients who met the inclusion and exclusion criteria in the screening phase uniformly received therapy (methotrexate and Yisaipu [recombinant human TNF receptor: Fc fusion protein, Guojian Pharmceutical company, China]) for 12 weeks. Those patients achieving clinical remission or low disease activity (DAS28 < 3.2) at 12 weeks were subsequently randomized into different treatment arms. Paired serum samples were collected at the time before (at baseline) and after 12 weeks’ therapy respectively, while RA disease activity were also evaluated. After uniformed treatment for 12 weeks, each RA patient was categorized as ACR20 responders, ACR50 responders, ACR70 responders, and ACR non-responders. ACR response criteria are widely used to assess and establish the improvement in tender or swollen joint counts along with improvement in three of the following five parameters: Acute phase reactant, Patient assessment, Physician assessment, Pain scale, and Disability/functional questionnaire. Achieving ACR20/50/70 means patients achieved at least 20/50/70% improvement in tender or swollen joint counts, as well as 20/50/70% improvement in three of the other five parameters. And those RA patients who achieve ACR 20/50/70 would have a significant decrease of disease activity. By using self-controlled design, we are able to catch the dynamic changes of the Cyr61 level along with RA disease activity.

Subjects with conditions known to influence Cyr61 levels were excluded, for instance, other autoimmune diseases, cancer, diabetes mellitus, infection, liver diseases, and coronary heart diseases.

This study was approved by the Institutional Medical Ethics Review Board of Peking University First Hospital (2014-785), and all clinical investigations were conducted according to the principles of the Declaration of Helsinki. Informed written consent was obtained from all study participants.

### Clinical assessments

Clinical data, including tender joint count (TJC), swollen joint count (SJC), erythrocyte sedimentation rate (ESR), C-reactive protein (CRP), patient global assessment (PGA), and evaluator global assessment (EGA), were obtained for all patients at all visits. Disease activity score based on 28 joint counts (DAS28) was calculated using either ESR (DAS28-ESR) or CRP (DAS28-CRP). The clinical disease activity index (CDAI) and simplified disease activity index (SDAI) were also calculated [16]. Active RA was defined as DAS28-ESR ≥ 3.2, or DAS28-CRP ≥ 3.2, or SDAI ≥11, or CDAI ≥10 in the corresponding scoring system. In the validation cohort, RA patients were regarded as responders when they met the ACR improvement criteria (ACR20/50/70 response, respectively) at 12 weeks after therapy. Patients who did not satisfy the ACR20 improvement criteria were defined as ACR non-responders.

### Enzyme-linked immunosorbent assay (ELISA)

Cyr61 concentration in undiluted serum samples were measured by enzyme-linked immunosorbent assay (ELISA) according to the manufacturer’s instructions (DY4055, R&D Systems, MN, USA). Each sample was tested in duplicate. The absorbance was measured at 450 nm, and serum Cyr61 concentration was calculated according to a standard curve.

### Statistical analysis

A Kolmogorov-Smirnov test of normality was performed for all variables. Quantitative data were presented as mean ± standard deviation (SD) if the data were normally distributed, or expressed as median and interquartile range (IQR) if the data did not follow Gaussian distributions. Categorical variables were described as percentages. In addition, quantitative data were analyzed using independent Student’s *t* test (for parametric data) or Mann-Whitney *U* test (for nonparametric data). Categorical data were compared using chi-squared test and Fisher’s exact test. One-way analysis of variance (ANOVA) was used to determine whether there were any statistically significant differences between the means of three or more independent groups and followed by Tukey’s post hoc test for pairwise comparisons. Spearman’s correlation coefficient was used to examine the relationship between the serum concentration of Cyr61 and disease activity. The discriminatory capacity of Cyr61 was assessed by receiver operating characteristic (ROC) curves based on data from the training cohort, and the best cutoff in terms of sensitivity and specificity was identified. In the validation cohort, the levels of Cyr61 before and after treatment were compared with Wilcoxon matched-pairs signed rank test. Logistic regression analysis was used to determine indicated factors for achieving ACR20 response. For all statistical analyses, *P* <  0.05 was considered as being statistically significant. Data analyses and graphing were conducted by SPSS 20.0 (SPSS Inc., Chicago, IL) and Prism software 6 (GraphPad Software, San Diego, CA).

## Results

### Demographics and clinical features of RA patients in the training cohort

In the training cohort, a total of 177 RA patients and 50 healthy controls were enrolled. There were no significant differences in demographics between RA patients and controls at the enrollment time. Approximately, the mean age was 52 years with 80% female and body mass index (BMI) of 21. Rheumatoid factor (RF) was present in 138 (77.97%) RA patients, and 148 (83.62%) patients were positive for anticitrullinated peptide antibodies (ACPA). The median disease duration of RA was 62 months. RA patients were further categorized according to their disease activity. There were 73 (41.24%) patients with moderate/high disease activity (DAS28-ESR ≥ 3.2, defined as active RA thereafter) and 104 (58.76%) patients with low disease activity/remission (DAS28-ESR < 3.2, defined as inactive RA thereafter). No significant difference was observed between these two groups of RA patients with respect to age, gender, BMI, disease duration, RF/ACPA positivity, and medication used. As expected, levels of CRP, ESR, TJC, SJC, PGA, EGA, and disease activity composite scores were higher in active RA patients compared to those in inactive RA patients. The demographics and clinical features of RA patients are shown in Table 1.

**Table 1:** Baseline clinical characteristics and Cyr61 concentration of RA patients in the training cohort

Variables	Inactive RA (*n* = 104)	Active RA (*n* = 73)	*P* value
Age (years)	51.97 ± 13.27	54.27 ± 15.96	0.158
BMI (kg/m^2^)	20.68 ± 5.19	22.19 ± 6.74	0.486
Gender (female, %)	81 (77.88%)	62 (84.93%)	0.332
Cyr61 concentration (pg/ml)	226.85 (152.50–339.96)	189.92 (129.58–254.47)	0.004
Disease duration (months)	60 (32–135)	64 (27–132)	0.68
RF-positive (*n*, %)	81 (77.88%)	57 (78.08%)	1.000
ACPA-positive (*n*, %)	88 (84.62%)	60 (82.19%)	0.832
Disease activity			
ESR (mm/h)	10 (5–18)	27 (17–47)	< 0.001
CRP (mg/l)	3.26 (1.90–5.55)	8.89 (4.18–18.70)	< 0.001
TJC (0–28)	0 (0–1)	6 (3–10)	< 0.001
SJT (0–28)	0 (0–0)	2 (1–4)	< 0.001
PGA (0–100)	10 (0–15)	50 (30–60)	< 0.001
EGA (0–100)	5 (0–10)	50 (30–60)	< 0.001
DAS28-ESR	1.92 (1.41–2.48)	4.64 (3.81–5.62)	< 0.001
DAS28-CRP	1.76 (1.50–2.26)	4.19 (3.20–5.21)	< 0.001
SDAI	2.15 (0.63–5.04)	18.30 (11.73–29.65)	< 0.001
CDAI	1.50 (0.10–4.38)	17.00 (11.00–27.00)	< 0.001
Medication in use (*n*, %)			
Methotrexate	82 (78.85%)	59 (80.82%)	0.850
Glucocorticoids	15 (14.42%)	17 (23.28%)	0.165
Leflunomide	7 (6.73%)	9 (12.33%)	0.287
Hydroxychloroquine	41 (39.42%)	25 (34.25%)	0.529
Sulfasalazine	6 (5.77%)	6 (8.22%)	0.553
TNF inhibitors	2 (1.92%)	4 (5.48%)	0.232

*RA* rheumatoid arthritis, *BMI* body mass index, *Cyr61* cysteine-rich 61, *RF* rheumatoid factor, *ACPA* anticitrullinated peptide antibodies, *ESR* erythrocyte sedimentation rate, *CRP* C-reactive protein, *TJC* tender joint count, *SJC* swollen joint count, *PGA* patient global assessment, *EGA* evaluator global assessment, *DAS28* disease activity score in 28 joints, *SDAI* simplified disease activity index, *CDAI* clinical disease activity index, *TNF* tumor necrosis factor

Values are presented as mean ± standard deviation or median (interquartile ranges), as applicable

*P* value means active RA vs. inactive RA

### Serum Cyr61 levels and its relationship with RA disease activity in the training cohort

**Fig. 1:**
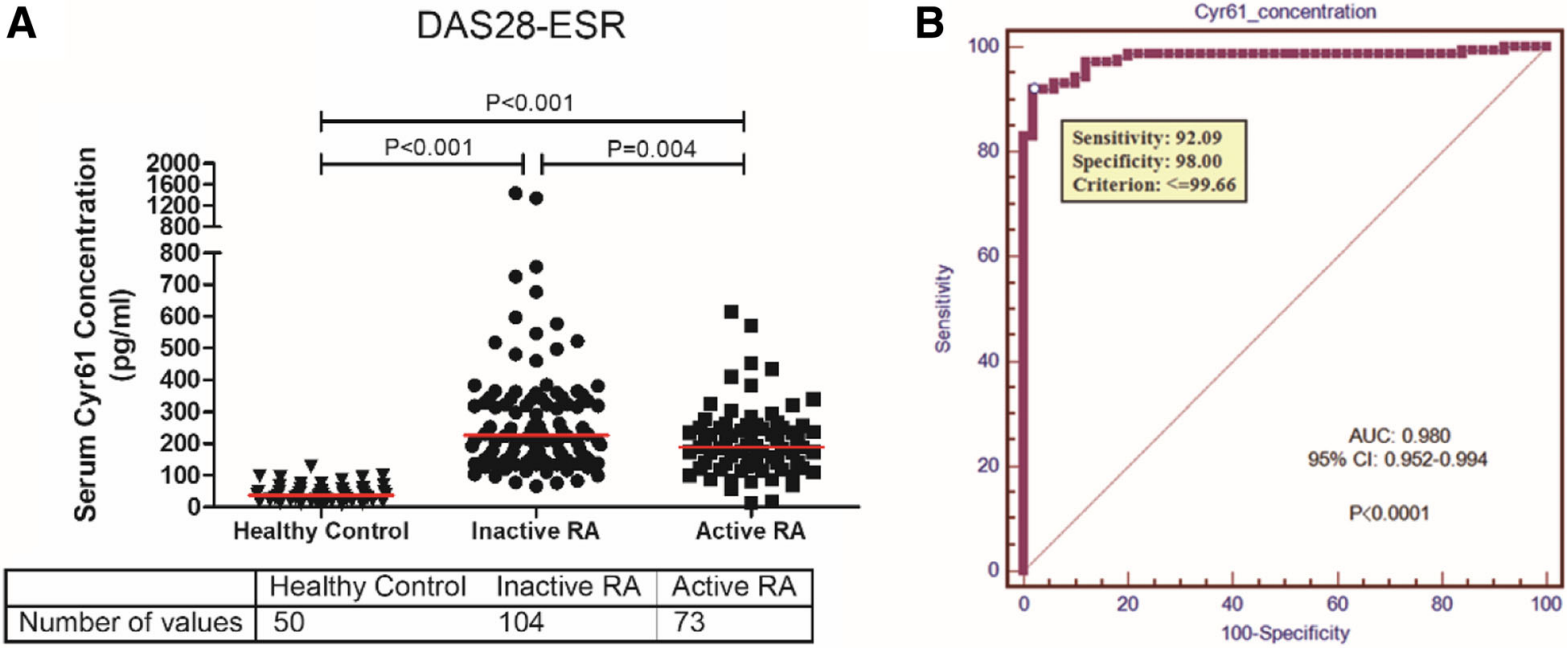
Serum Cyr61 concentrations and diagnostic performance of Cyr61 in participants in the training cohort. **a** Levels of serum Cyr61 in healthy controls and RA patients (classified into inactive RA group and active RA group as evaluated by DAS28-ESR ≥ 3.2). **b** Receiver operating characteristic (ROC) curve to determine the best threshold for Cyr61 to discriminate RA and healthy controls. The red horizontal solid line represents a median value

Serological levels of Cyr61 were remarkably higher in 177 RA patients compared to healthy controls (median [IQR] 211.57 [140.66–319.01] vs. 37.24 [22.82–56.16], *P* <  0.001) (Fig. 1a). An ROC curve showed superior ability of serum Cyr61 concentration in discriminating RA from healthy controls with an area under curve (AUC) of 0.980 (95% CI, 0.952–0.994, *P* <  0.001) (Fig. 1b). The optimal cutoff value of Cyr61 was 99.66 pg/ml, with the sensitivity of 92.09%, the specificity of 98.00%, the positive predictive value (PPV) of 99.39%, and the negative predictive value (NPV) of 77.78%.

**Fig. 2:**
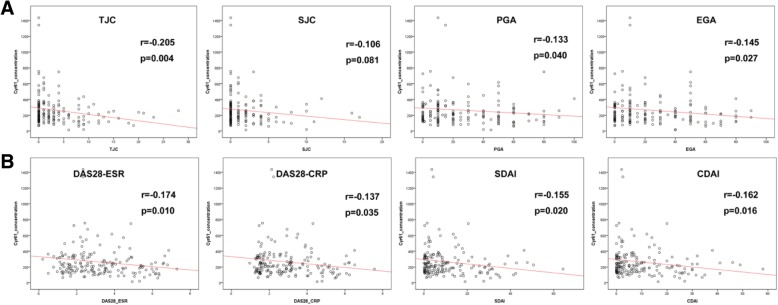
Scatterplots showing correlations between Cyr61 levels with disease activity measures in RA patients in the training cohort. **a** Left to right: tender joint count (TJC), swollen joint count (SJC), patient global assessment (PGA), evaluator global assessment (EGA). **b** Left to right: DAS28-ESR, DAS28-CRP, simplified disease activity index (SDAI), clinical disease activity index (CDAI). *R* value represents Spearman’s correlation coefficient

Surprisingly, we found that serum Cyr61 concentration in RA patients with remission/low disease activity was significantly higher than in RA patients with moderate/high disease activity (median [IQR] 226.85 [152.50–339.96] vs. 189.92 [129.58–254.47], *P* = 0.004) (Table 1 and Fig. 1a). Similar trends were also observed and confirmed in RA patients with different disease activity defined by DAS28-CRP, SDAI, and CDAI (Additional file 1: Figure S1). We further categorized TJC and SJC, the important components of the composite scoring system, as 0 joint, 1 joint, 2–4 joints, and ≥ 5 joints. All these joint count categories were in general associated with high Cyr61 levels relative to healthy controls, with the highest Cyr61 concentration in patients with nil TJC/SJC. With the increasing number of TJC/SJC, Cyr61 concentration decreased. Compared to the 0 joint group, the Cyr61 concentration was significantly lower in the ≥ 5 joints group (Additional file 1: Figure S2). These indicated that Cyr61 expression was associated with disease activity in RA. Further, Spearman correlation analysis showed that the Cyr61 level was inversely correlated with composite disease activity scores and almost all of their components in statistic, including DAS28-ESR (*r* = − 0.174, *P* = 0.010), DAS28-CRP (*r* = − 0.137, *P* = 0.035), SDAI (*r* = − 0.155, *P* = 0.020) and CDAI (*r* = − 0.162, *P* = 0.016), TJC (*r* = − 0.202, *P* = 0.004), PGA (*r* = − 0.133, *P* = 0.040), and EGA (*r* = − 0.145, *P* = 0.027) (Fig. 2).

### Serum Cyr61 levels in RA patients in the validation cohort before and after treatment

**Fig. 3:**
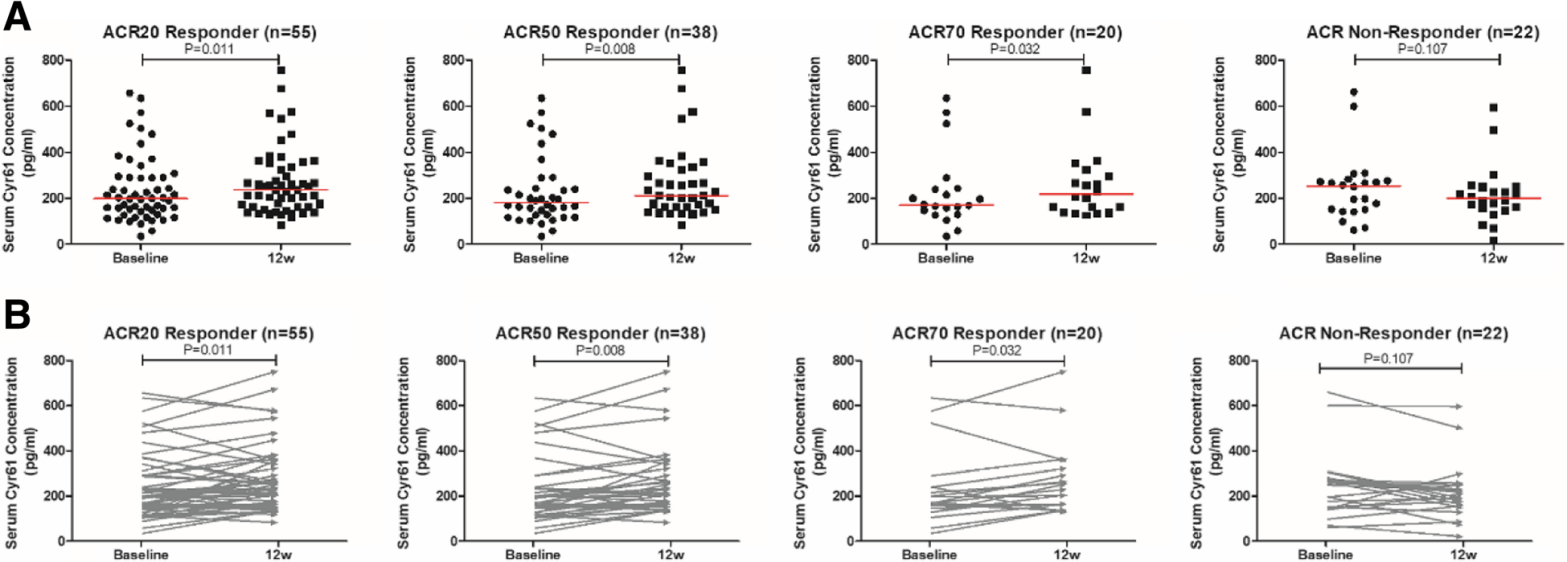
Serum Cyr61 levels in RA patients in the validation cohort before and after treatment. **a** Left to right: scatter plots showing the levels of Cyr61 in ACR20 responders, ACR50 responders, ACR70 responders, and ACR non-responders before and after treatment. **b** Left to right: before-after plots showing the levels of Cyr61 in ACR20 responders, ACR50 responders, ACR70 responders, and ACR non-responders before and after treatment (in correspondence with **a**). The red horizontal solid line represents a median value

Considering the high heterogeneity of RA individuals may influence the expression of Cyr61, we then investigated the relationship between Cyr61 and disease activity in our validation cohort based on a randomized and controlled clinical trial (ClinicalTrials.gov identifier: NCT02320630). Seventy-seven active RA patients (DAS28-CRP > 3.2) who received methotrexate in combination with TNF inhibitor therapy for 12 weeks were enrolled in the validation cohort. The mean age of these RA patients was 55.56 years old with disease duration of 93 months. After 12 weeks of treatment, there was a significant reduction in disease activity score (DAS28-ESR from 5.39 ± 1.31 to 3.34 ± 1.39, *P* <  0.05; DAS28-CRP from 4.92 ± 1.28 to 3.12 ± 1.21, *P* <  0.05). Fifty-five (71.43%) patients successfully achieved ACR20 response, 38 (49.35%) patients achieved ACR50 response, and 20 (25.97%) patients achieved ACR70 response, while 22 (28.57%) RA patients were ACR non-responders (Table 2). Serum samples of all patients were collected at baseline and 12 weeks after the treatment respectively. Wilcoxon matched-pairs signed rank test showed the levels of Cyr61 in ACR20 responders were significantly upregulated at 12 weeks from baseline (median [IQR] 199.73 [142.38–290.33] vs. 236.21 [174.17–338.14], *P* = 0.011). Similarly, there was also a significant increase in Cyr61 concentration in ACR50 or ACR70 responders. On the contrary, for those ACR non-responders, no significant change of Cyr61 levels from baseline to 12 weeks, and even a trend to be lower after treatment, was observed (Fig. 3). Those who were treated effectively (disease activity decreased) would show a significant increase of the Cyr61 level. These further strengthened the inverse correlation of Cyr61 level with disease activity in RA patients.

**Table 2:** Baseline clinical characteristics and treatment response of RA in the validation cohort

Variables	RA patients (*n* = 77)
Age (years)	55.56 ± 12.53
Gender (female, %)	60 (77.92%)
BMI (kg/m^2^)	21.47 ± 5.87
Disease duration (months)	93 (42–184)
RF-positive (*n*, %)	57 (74.03%)
ACPA-positive (*n*, %)	62 (80.52%)
ACR20 responders (*n*, %)	55 (71.43%)
ACR50 responders (*n*, %)	38 (49.35%)
ACR70 responders (*n*, %)	20 (25.97%)
ACR non-responders (*n*, %)	22 (28.57%)

*BMI* body mass index, *RF* rheumatoid factor, *ACPA* anticitrullinated peptide antibodies, *ACR 20/50/70* American College of Rheumatology 20%/50%/70% improvement criteria

Whether the increase of Cyr61 level had an independent association with ACR treatment response was worthy of exploration. Further, univariate and multivariate logistic regression analyses were conducted and demonstrated that increase of serum Cyr61 and younger age were independent indicators for achieving ACR20 response, adjusted by gender, BMI, RF/ACPA positivity, and disease duration (Table 3 and Additional file 1: Table S1). Moreover, an increased Cyr61 level was dichotomized into high increase and low increase based on the median value of the variable. It was further revealed by univariate and multivariate logistic regression analyses that a high increase of the Cyr61 level was independently associated with ACR20 response with OR 2.988 (95% CI 1.020–8.754, *P* = 0.029) (Table 3 and Additional file 1: Table S1). Taken together, it was indicated that increase of serum Cyr61 (both dichotomic variable and continuous variable) was an independent indicator for achieving ACR20 response criterion (disease activity decreased) after adjusting potential confounders.

**Table 3:** Independent associated factors to predict ACR20 response after 12 weeks using multivariate logistic regression analysis

Variables	Odds ratio	95% confidence interval	*P* value
Analysis set 1			
Age at baseline (per year increase)	0.939	0.887–0.997	0.029
Increased Cyr61 (per unit increase)	1.008	1.002–1.014	0.005
Analysis set 2			
Age at baseline (per year increase)	0.951	0.905–0.999	0.046
Increased Cyr61 (high increase vs. low increase)	2.988	1.020–8.754	0.029

## Discussion

Cyr61 is a secreted matricellular protein, encoded by a growth factor-inducible immediate-early gene. It is transcriptionally activated within minutes of stimulation by “injury” stimuli (especially inflammation), whereas the encoding gene is expressed at low levels in quiescent cells [3]. Numerous preclinical studies have uncovered a pivotal role of Cyr61 in RA pathogenesis. Overexpression of Cyr61 in RA synovial fluids or synovial tissues contributed to FLS proliferation via an autocrine or paracrine manner [7, 9]. And Cyr61 secreted by FLS subsequently stimulated the production of various proinflammatory mediators (IL-17, IL-6, IL-8, and IL-1β) through Akt/NF-κb and MAPK signaling pathways and thereby participated in the pathologic mechanism in RA [7–9, 17]. In the present study, we demonstrated that serum Cyr61 was remarkably elevated in RA patients compared to healthy controls, which was consistent with previous preclinical studies showing overexpressed Cyr61 in RA synovial fluids as well as peripheral blood mononuclear cells. The conservative expression of Cyr61 in healthy control determined its very low level in serum (median [IQR] 37.24 [22.82–56.16]). ROC analysis revealed a strong overall performance characteristic of serum Cyr61 concentration in identifying RA patients from healthy controls, with an area under curve of 0.980 (95% CI 0.952–0.994, *P* <  0.001).

Although there are several publications about Cyr61 in RA, the relationship between Cyr61 levels with RA disease activity has never been investigated. In our training cohort, we stratified our RA patients into moderate/high disease activity group and remission/low disease activity group. Intriguingly, we found that a serum Cyr61 level was inversely correlated with RA disease activity. This inverse correlation was also confirmed in RA patients defined by other disease activity scoring systems (DAS28-CRP, SDAI, and CDAI). When RA patients were stratified by numbers of TJC and SJC, the Cyr61 levels were observed highest in nil TJC/SJC group and decreased in patients with increasing number of TJC/SJC. Spearman correlation analysis revealed that Cyr61 levels were negatively correlated with almost all disease activity indices in statistic. Considering the possible limitation of cross-sectional study, we evaluated the expression of Cyr61 in our validation RA cohort to further confirm aforementioned findings. All these active RA patients received methotrexate in combination with TNF inhibitor therapy for 12 weeks. At the end of 12 weeks, the level of Cyr61 was significantly increased from baseline in ACR20 responders (mean increase 30.1%), ACR50 responders (mean increase 35.7%), and ACR70 responders (mean increase 41.0%), while no significant change was found in non-responders. By far, we concluded with confidence that the serological Cyr61 level was inversely correlated with RA disease activity. A similar case, in colorectal cancer, it was reported that mRNA expression of Cyr61 was significantly higher than normal colons, and the expression was also relatively low in patients with more advanced cancer [18]. Another study in lung cancer by Mori et al. demonstrated that patients with low Cyr61 expression was clinically faster progressing than those with high Cyr61 expression [19]. A previous multi-center study with the aim of exploring the potential value of Cyr61 in systemic lupus erythematosus-associated pulmonary arterial hypertension patients also revealed that a higher Cyr61 level predicted better survival than those with a lower Cyr61 level [20].

The possible interpretations to this negative correlation would be intricate and partially owing to the functional diversity of Cyr61. As an evolutionary ancient matricellular protein, Cyr61 acted primarily through direct binding to distinct integrin receptors in a cell-specific manner to regulate diverse cellular responses, even opposed [3, 21]. As mentioned above, a multitude of studies uncovered the proinflammatory properties of Cyr61. Nevertheless, accumulating high-quality evidence demonstrated anti-inflammatory and protective features of Cyr61. For example, a proof-of-concept study used a gene-transfer approach to overexpress Cyr61 in experimental autoimmune myocarditis mice and unexpectedly found Cyr61 overexpression reduced the disease scores and cardiac immune cell infiltrations. Long-term stimulation with Cyr61 ameliorated inflammatory processes by inhibiting immune cell trafficking in vitro and in vivo [12]. By using Cyr61 gene-modified mice, Professor Lau uncovered a critical role of Cyr61 in triggering efferocytosis of neutrophils by macrophages to promote resolution of inflammation in skin wounds [14]. The team further demonstrated for the first time that Cyr61 played critical roles in promoting recovery and mucosal healing in colitis. Administration of exogenous Cyr61 accelerated mucosal restitution from colitis in both wild type and Cyr61 mutant mice, underscoring a therapeutic potential for Cyr61 in inflammatory bowel disease (IBD) [22]. Of note, inflammatory bowel disease and RA share important pathogenesis mechanisms, at least in part, through the contribution of the Th1/Th2 cytokine balance [23]. Whether Cyr61 plays a protective role in RA just as it does in IBD needs to be further assessed by using more precise animal experiments and clinical studies with a larger sample size. Nevertheless, the dramatic upregulation of Cyr61 expression in RA has implicated its role in arthritis development and progression. Despite we did the analysis in our “validation cohort” at baseline and at 12 weeks of treatment, it should also be noted that a better analysis would be a longitudinal analysis with multiple measures that could evaluate the Cyr61 level and its correlation with RA disease activity dynamically.

To the best of our knowledge, this is the first study to investigate the serum Cyr61 level and its association with disease activity in RA patients. By using a training cohort as well as a validation cohort, we confirmed the serum Cyr61 levels were inversely correlated with RA disease activity. It should be noted that the exact mechanisms underlying this phenomenon had not been explored in the study. One can suppose that, depending on Cyr61 expression pattern in distinct cell types and selected times, the balance between its dual permissive and inhibitory activities on different cells can lead to either beneficial or deleterious effects upon inflammatory processes. The present clinical study provides important implications for Cyr61 in RA, but understanding how Cyr61 protein functions in RA pathogenesis requires further investigation.

## Conclusions

Serum Cyr61 levels were remarkably increased in RA patients compared with healthy controls. Intriguingly, the level of Cyr61 was inversely correlated with RA disease activity. Therefore, Cyr61 has great potentials to be a biomarker for monitoring RA disease activity, predicting treatment response, and being a therapeutic target. Prospective clinical studies as well as basic researches are needed in the future.

## Additional file

Additional file 1: **Table S1.** Univariate logistic regression analysis for associated factors to predict ACR20 response after 12 weeks in validation cohort. **Figure S1.** Serum Cyr61 concentrations in RA patients in the training cohort stratified by other disease activity score systems. **Figure S2.** Serum Cyr61 concentrations in RA patients in the training cohort stratified by TJC and SJC. (DOCX 347 kb)

## Abbreviations

ACPA: Anticitrullinated peptide antibodies
ACR: American College of Rheumatology
BMI: Body mass index
CDAI: Clinical disease activity index
CRP: C-reactive protein
Cyr61: Cysteine-rich 61 (also called CCN1)
DAS28: Disease activity score in 28 joints
DMARDs: Disease-modifying antirheumatic drugs
EGA: Evaluator global assessment
ELISA: Enzyme-linked immunosorbent assay
ESR: Erythrocyte sedimentation rate
FLS: Fibroblast-like synoviocyte
IL: Interleukin
PGA: Patient global assessment
RA: Rheumatoid arthritis
RF: Rheumatoid factor
SDAI: Simplified disease activity index
SJC: Swollen joint count
TJC: Tender joint count
TNF: Tumor necrosis factor
