# Supplementary material for: Cysteine-rich 61 (Cyr61): a biomarker reflecting disease activity in rheumatoid arthritis
Source: Arthritis Res Ther. 2019 May 21;21:123. doi: 10.1186/s13075-019-1906-y (PMC6528265; doi:10.1186/s13075-019-1906-y)

**Additional file 1**

**Table S1**. Univariate logistic regression analysis for associated factors to predict ACR20 response after 12 weeks in validation cohort

| **Variables** | Odds ratio | 95% confidence interval | P value |
| --- | --- | --- | --- |
| Age at baseline (per year increase) | 0.949 | 0.903-0.998 | **0.041** |
| Disease duration (per year increase) | 1.021 | 0.955-1.092 | 0.542 |
| Gender (female vs. male) | 0.718 | 0.206-2.504 | 0.603 |
| BMI (per unit increase) | 0.589 | 0.983-1.232 | 0.502 |
| RF positivity | 2.833 | 0.738-10.878 | 0.129 |
| ACPA positivity | 3.095 | 0.637-15.043 | 0.161 |
| Increased Cyr61 (per unit increase) | 1.008 | 1.002-10.014 | **0.009** |
| Increased Cyr61 (high increase vs. low-increase) | 2.981 | 1.049-8.477 | **0.040** |

**Figure S1** Serum Cyr61 concentrations in RA patients in the training cohort stratified by DAS28-CRP, SDAI (simplified disease activity index) and CDAI (clinical disease activity index). The red horizontal solid line represents median value.


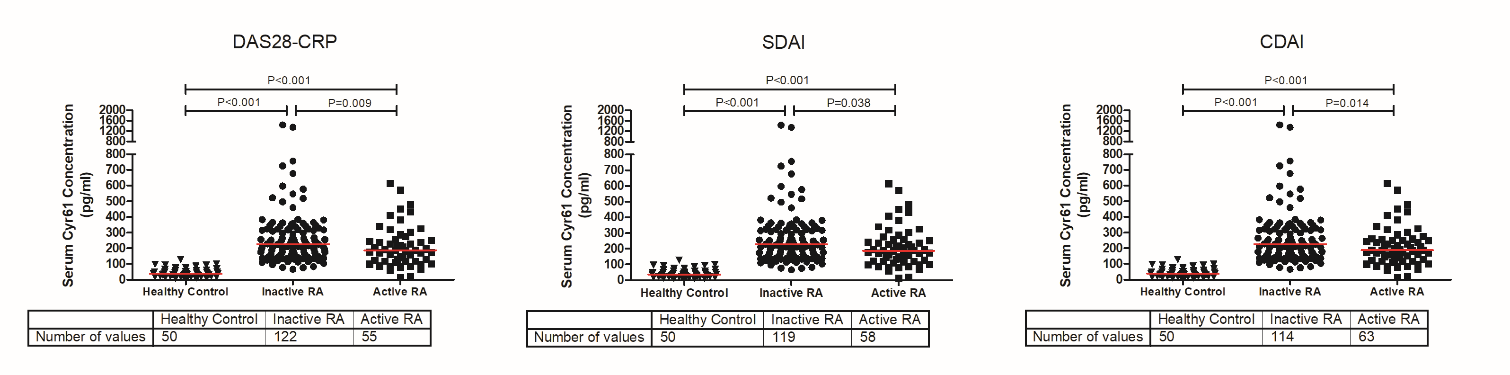


**Figure S2** Serum Cyr61 concentrations in RA patients in the training cohort stratified by tender joint count (A) and swollen joint count (B). The red horizontal solid line represents median value.


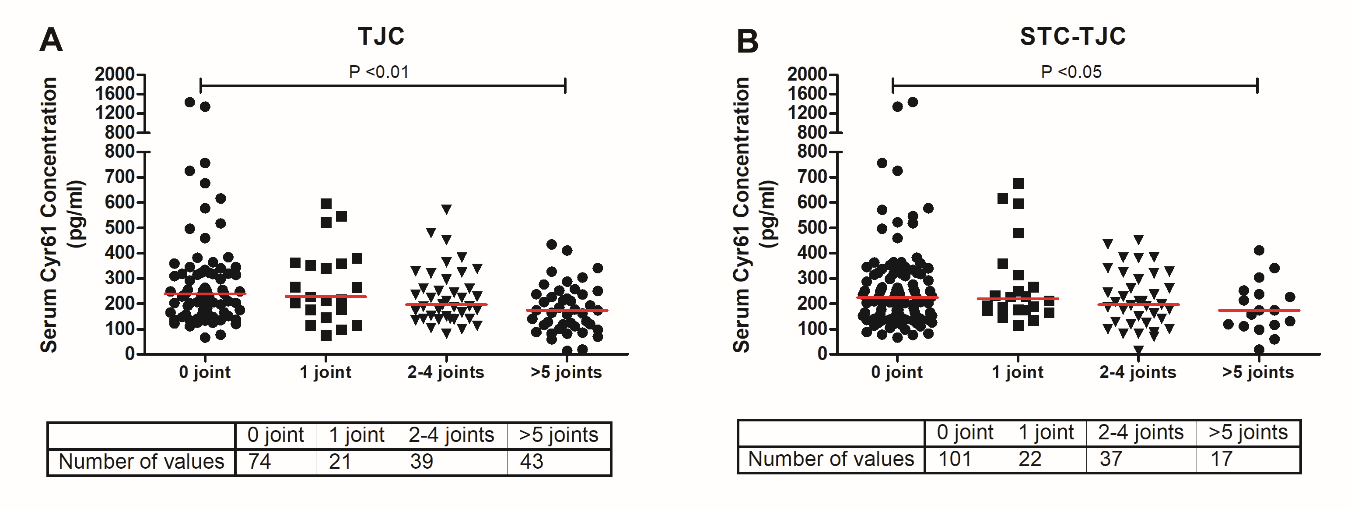

Supplement: Supplementary file 1 — Table S1. Univariate logistic regression analysis for associated factors to predict ACR20 response after 12 weeks in validation cohort. Figure S1. Serum Cyr61 concentrations in RA patients in the training cohort stratified by other disease activity score systems. Figure S2. Serum Cyr61 concentrations in RA patients in the training cohort stratified by TJC and SJC. (DOCX 347 kb) [file 13075_2019_1906_MOESM1_ESM.docx]
